# EDITOR'S COMMENTS

**Published:** 2009

Temporomandibular joint is a complex and precisely integrated bilateral joint structure. It is a synovial, bicondylar, diarthrodial joint more specifically classified as ginglymus or ginglymoarthrodial joint, that shows both sliding and hinge movements. The two condylar joints on each side act independently or synchronously.

The temporomandibular joint consists of the mobile condyloid process of the mandible which articulates with the fixed glenoid fossa of the temporal bone. Between the condylar process and the glenoid fossa lies an interposed cartilaginous disc. The disc provides a stable platform for the rotational and gliding movements of the joint. It also acts as a shock absorber. The disc divides the temporomandibular joint into an upper temporodiscal and a lower condylodiscal compartments. The condylodiscal compartment is associated with ‘rotation’ or ‘hinge’ movement. The forward ‘translation’ movement takes place in the upper temporodiscal compartment in which the condyle moves downwards and forwards towards and over the articular eminence. During jaw opening the condyle first rotates as the mandible travels from its Retruded Contact Position to the point of Centric Occlusion. This is followed by downward and forward translocation of condyle wich takes the mandible from Centric Occlusion to the point of maximal mandibular opening. This is called Posselt's envelope of mandibular motion. During sideways movement, the condyle towards the movement rotates with slight lateral shift – Bennett Shift. The contralateral condyle shows corrosponding forward translation and rotation movements.

Considering the complexity of the anatomy and physiology of this joint, whether a simple ball & socket assembly can replace this joint to perfection is certainly debatable. Yes a costo-chondral graft, which has been traditionally used to replace the condyle is mechanically no better but it has many advantages – it can grow like the opposite condyle, although not equally at times, and in a paediatric patient is any day better than a prosthesis for this very reason. Furthermore it is body's own tissue and once the graft it taken up at the recipient site it will be there life long; prosthesis, no matter how well fitting, will certainly not tolerate the masticatory forces for life. So though we appreciate the novel effort put in by the author, we do not agree with his choice of patient and we would like him to report his long term follow up in the pages of our Journal.

